# Review of the Early Diagnoses and Assessment of Rejection in Vascularized Composite Allotransplantation

**DOI:** 10.1155/2013/402980

**Published:** 2013-02-13

**Authors:** Ravi Starzl, Gerald Brandacher, W. P. Andrew Lee, Jaime Carbonell, Wensheng Zhang, Jonas Schnider, Vijay Gorantla, Stefan Schneeberger, Xin Xiao Zheng

**Affiliations:** ^1^Language Technologies Institute, Carnegie Mellon University, Pittsburgh, PA 15213, USA; ^2^Department of Plastic and Reconstructive Surgery, University of Pittsburgh, Pittsburgh, PA 15261, USA; ^3^Department of Plastic and Reconstructive Surgery, Johns Hopkins University School of Medicine, 720 Rutland Avenue, Baltimore, MD 21287, USA; ^4^Center of Operative Medicine, Department of Visceral, Transplant and Thoracic Surgery, Innsbruck Medical University, Anichstraße 35, 6020 Innsbruck, Austria; ^5^Research Center for Translational Medicine, Shanghai East Hospital, Tongji University, Shanghai 200120, China

## Abstract

The emerging field of vascular composite allotransplantation (VCA) has become a clinical reality. Building upon cutting edge understandings of transplant surgery and immunology, complex grafts such as hands and faces can now be transplanted with success. Many of the challenges that have historically been limiting factors in transplantation, such as rejection and the morbidity of immunosuppression, remain challenges in VCA. Because of the accessibility of most VCA grafts, and the highly immunogenic nature of the skin in particular, VCA has become the focal point for cross-disciplinary approaches to developing novel approaches for some of the most challenging immunological problems in transplantation, particularly the early diagnoses and assessment of rejection. This paper provides a historically oriented introduction to the field of organ transplantation and the evolution of VCA.

## 1. Organ Transplantation

The concept of replacing organs or limbs that have become diseased or damaged is a deeply rooted human dream so old that it has been incorporated into our mythology in chimeric beings like the Hindu Ganesha [1]. The oldest recorded attempted transplant was the use of skin from a donor to conduct a reconstructive rhinoplasty on another man, performed by the classical Indian surgeon Sushruta, sometime between 1000 and 600 BCE [2–4]. Throughout the ages surgeons have attempted transplantation time and again, but it was not until key contributions from Medawar, Brent, and Billingham at the turn of the 20th century that real progress in understanding the biology underlying host-allograft interactions was made [5–7]. At approximately the same time, important insights into the circulation and role of lymphocytes in immunologic response were being made [8–12]. This essential work came on the heels of important early descriptions of lymphocyte activity in inflammation [13–16].

As the scientific foundations for transplant biology rapidly evolved, the first successful kidney transplant between identical twins was conducted in 1954 [17, 18]. Although a surgical success, little immunologic information was generated because the transplant was not an allograft (or homograft). The monozygotic twins were genetically identical and therefore shared the same major histocompatibility complex (MHC). Rejection rarely occurs in such cases. The identical twin transplant of 1954 was an isograft, immunologically closer to an autograft than an allograft, and the potent issues of allogenicity were left unresolved. It would not be until the 1960s that appreciable graft survival was achieved in MHC mismatched patients [19–22].

Throughout the 1960s and 1970s, attempts to control rejection included irradiation of the recipient to neutralize the host immune system [12, 23–27], the administration of azathioprine [19–22], and eventually treatment with antilymphocyte globulin (ALG/ALS) [28–32]. Although these were shown to have beneficial effects on graft survival, morbidities were extensive [33–35], rejection was still a threat [36], and graft-versus-host disease would sometimes overtake patients [37–44].

With the arrival of cyclosporine in the late 1970s, a new era in the clinical viability of transplantation as a therapeutic intervention dawned. Significant improvements in outcome and graft survival were achieved first in liver [45], then in kidney [46] patients. A new class of immunosuppressant cyclosporine was powerful enough to provide the high levels of immunosuppression required for managing transplants, with fewer of the morbidities associated with prior treatment regimens.

However, these improvements came with a price. Cyclosporine was shown to be nephrotoxic over time [47–49], and care still had to be taken to avoid the morbidities associated with a suppressed immune system, such as infection [50]. Despite these drawbacks, the level of clinical improvement cyclosporine offered over previous methods was very compelling, and cyclosporine fueled much of the explosive growth in transplantation during the 1980s and beyond [51–53].

In late 1987 a report from Japan introduced FK-506 (Tacrolimus) as a new and potent immunosuppressive agent [54]. Additional studies rapidly followed in more animal models, confirming FK-506's effectiveness in suppressing and rescuing grafts from rejection [55–61]. Synergistic effects with cyclosporine were also observed [56, 62]. The potency of FK-506, and its synergistic effects with other drugs, would open the door for future therapeutic strategies to leverage immunosuppression dosage as a controller for modulating the tolerance/rejection balance in transplants [63].

The search for cyclosporine's mechanism of action began almost immediately after it was shown to have clinical promise, but it was not until after the introduction and clinical adoption of FK-506 in the early 1990s that both FK-506 and cyclosporine were discovered to inhibit the calcineurin phosphatase pathway [64–67]. Further studies rapidly elucidated additional mechanism details in subsequent years.

Although mainstream clinical practice had vigorously adopted high-dose combination immunosuppression therapy as the treatment of choice because of the specter of rejection, in 1992 the notion that more immunosuppression was not necessarily better emerged. A group of patients were discovered to have become chimeric or developed tolerance towards their allograft [68], helping to elucidate the fact that allografts carried passenger leukocytes that conducted an immune response against the host; much as the host carries out an immune reaction against the allograft [69]. This became known as the double-immune response or clonal exhaustion and deletion [70]. Further investigation of these cases revealed that moderate levels of immunosuppression, carefully timed and tailored to each individual, were at least partially successful in eliminating patient dependence on lifelong immunosuppression [71]. Prior to these observations the clinical view was that the immune response needed to be quashed as early and completely as possible, in order to prevent the leviathan of rejection from emerging. However after the chimeric patients were discovered, the door to the consideration of more nuanced application of immunosuppression was opened. 

Organ transplantation has evolved from an essentially nonexistent field to one of the most prominent disciplines in medicine over the last sixty years.

## 2. Vascularized Composite Allotransplantation

In 1998 the first human hand transplant under current clinical standards of immunosuppression was conducted, making vascularized composite allotransplantation (VCA) a performed clinical reality. Over the past decade it has become a treatment option for the many patients suffering from complex tissue injuries or defects not amenable to conventional reconstruction [72]. More than 60 hand/forearm and most recently arm transplants as well as 90 hands and over 20 face transplants performed throughout the world have also shown that allograft survival with good functional outcomes can be routinely achieved after VCA [73–77]. However, despite the fact that surgical procedures and functional outcomes are highly successful, the need for long-term and high-dose immunosuppression to enable graft survival and to treat/reverse acute rejection episodes are the remaining and pace-limiting obstacles to widespread application [78, 79]. The toxicity profile of such drug treatment is considerable and includes serious side effects, such as opportunistic infections, malignancy, and end organ damage [80–83]. 

VCA recipients are unique in that they undergo a transplant procedure for what is considered to be a nonlife-threatening condition. Therefore, there is a critical need to develop immunosuppression minimization strategies to reduce the risks of chronic immunosuppression.

The skin is the principal target of rejection after VCA transplantation, making it an obstacle to tolerance induction or minimizing immunosuppression. On the other hand, due to its external location, the skin provides a unique clinical opportunity for monitoring, early diagnosis, prevention, and treatment of VCA rejection, including the possibility of therapies applied directly/topically to the skin. 

Acute rejection in hand transplantation appears with maculopapular skin lesions, which can be limited to a small area of the skin or can spread over large parts of the transplant [74, 75, 84–87]. 

Clinical macroscopic manifestations can range from mild pink discoloration or erythema to lichenoid papules, edema, and onychomadesis. The main histological feature of acute rejection is a mononuclear cell infiltrate. It first appears in the perivascular space of the dermis and then spreads to the interface between dermis and epidermis and/or adnexal structures. A perivascular, cellular infiltrate within the epidermis is typical for a moderate grade of rejection with the immunologic response reaching the outermost layer. If rejection is not successfully treated at that stage, necrosis of single keratinocytes can be observed, resulting in focal dermal-epidermal separation and significant graft damage [84, 86, 87]. If rejection progresses further, necrosis and loss of the epidermis, as the ultimate stage of skin rejection, are considered irreversible. However, very limited information is available on the involvement of components other than the skin in this acute rejection process [86]. The histological findings in VCA patients are in line with results from experimental studies indicating that the skin is highly immunogenic and hence the primary/sentinel target for rejection. This is further substantiated by the fact that immunological tolerance can be achieved towards all components of a VCA experimentally except the skin. It was also shown that skin alterations in a VCA are not exclusively limited to alloimmune-mediated injury. The clinical and histopathological features of immune-related and nonrejection processes are potentially overlapping or may coincide with acute rejection. The underlying mechanisms are largely unknown and represent a current major clinical challenge in differentiating between acute rejection and other forms of skin inflammation.

## 3. Cytokines in the Study of Skin Rejection

Skin rejection is becoming increasingly useful as a platform to study rejection because it is easy to access and can be monitored more consistently than internal organs during the process of rejection. Because of its high immunogenicity skin is a VCA that is prone to frequent and sudden episodes of rejection, much more so than other tissues such as muscle, making it a clinically important tissue to investigate from the perspective of VCA. Insight and understanding of the dynamics of rejection in skin will likely be elucidative for other tissues and lead to a more complete picture of immune system function under conditions of rejection.

**Figure 1:**
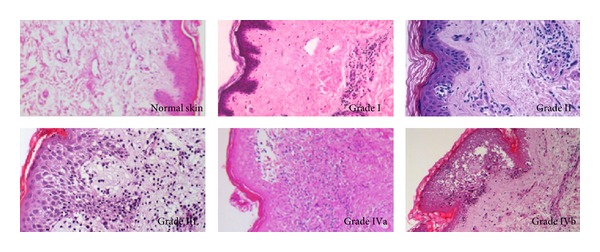
Banff Grading of Acute Skin Rejection in VCA; Allograft histology rejection grades. Grade 0: no or rare inflammatory cells, Grade I: mild perivascular infiltration. No involvement of overlying epidermis, Grade II: moderate. Perivascular inflammation with/without mild epidermal or adnexal involvement (limited to spongiosis and exocytosis). No epidermal dyskeratosis or apoptosis, Grade III: dense inflammation and epidermal involvement with apoptosis, dyskeratosis, and/or keratinolysis, Grade IV: necrotizing acute rejection. Frank necrosis of epidermis or other skin structures.

The Banff 97 Working Classification of Renal Allograft Pathology [88] provided a uniform basis for the grading rejection in allograft biopsies. It has been subsequently updated most recently by Banff 07 Classification of Renal Allograft Pathology: Updates and Future Directions [89]. Grading schemes relevant to skin and VCA were also defined in The Banff 2007 Working Classification of Skin-Containing Composite Tissue Allograft Pathology (Figure 1) [85].

Interestingly, in our recent unpublished study, in a rat hind limb allograft model we observed a differential rejection pattern in the animals receiving a long-last form of IL-2, IL-2/Fc fusion protein, in combination with antilymphocyte serum and cyclosporine A. Despite all animals undergoing early acute rejection, approximately 55% of them spontaneously recovered and went on to long-term survival for more than 200 days. Moreover, the cytokine and FoxP3 gene expression profiles from the skin biopsy at the earliest sign of rejection revealed a significant increased ratio of FoxP3 expression versus Granzyme, IFN-*γ*, and Perforin in the animals that spontaneously recovered (benign rejection) as against the animals who had a lower FoxP3 expression that went onto grade 4 rejection (progressive rejection). It suggested that, based on cytokine gene expression profiles from skin biopsy at the earliest sign of rejection, it may be possible to predict the ultimate course of the rejection and provide evidence for a proper treatment (paper in preparation).

## 4. Similarities in Early Skin Rejection and Other Sources of Skin Inflammation

Skin rejection in VCA is presented with erythematous macules that may progress if not treated to infiltrated scaly violaceous lichenoid papules covering the complete surface of the graft [90]. These alterations are not specific for rejection and may mimic inflammatory dermatoses. Kanitakis et al. emphasized the diagnostic challenges in early or mild skin rejection. Early rejection (grades 1 and 2) can be especially difficult to differentiate from contact dermatitis, insect bites, or dermatophyte infections. It is notable that histologic lesions such as eosinophilia, leukocytoclastic vasculitis, and demonstration of infectious antigens can indeed lend specificity to pathologic diagnoses. While the geographic limitation of lesions to the skin of the allograft can be an important and helpful hint, atypical cases of skin rejection with regard to the anatomical site, progression, or the clinical manifestation have been described [91] and the location alone cannot be considered proof. Early and accurate diagnoses, however, are critical to either prevent progression of rejection or incorrect treatment of the patient. 

Parallels between acute skin rejection and inflammatory dermatoses (e.g., contact dermatitis, psoriasis, and atopic dermatitis) also exist on the molecular and cellular levels. Allergic contact dermatitis, for example, is a T-cell-mediated-delayed-type hypersensitivity reaction that occurs upon hapten challenge in sensitized individuals [92]. Therefore, the differentiation mainly based on histological and macroscopic criteria can be difficult. It has been demonstrated that T cells (CD4+ and CD8+ cells) are critical effectors and that elements of the innate immune system (e.g., natural killer cells) may play a key role [93]. Epidermal Langerhans cells as the most powerful antigen presenting cells in skin as well as keratinocytes are regulating this inflammatory process. Cytokines derived from Langerhans cells (e.g., IL-12) and from T-cells (IFN-gamma, IL-4, and IL-10) play a pivotal role in the induction and initiation of this common skin disease [92, 94]. 

**Figure 2:**
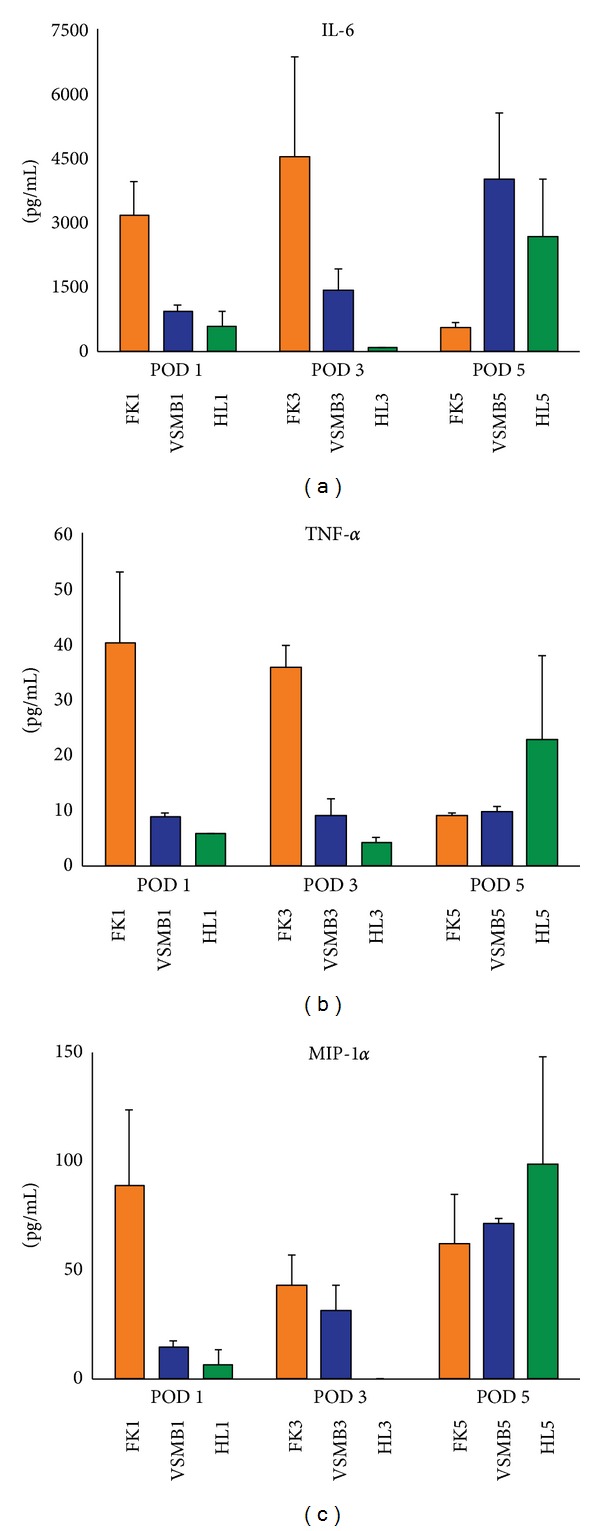
IL-6, TNF*α*, and MIP-1*α* are highly expressed from fullness skin (FS) allografts in comparison with that from hind limb (HL) and vascularized skin muscle bone (VSMB) allograft at POD 1 and POD 3.

In recently collected unpublished data, cytokine expression patterns associated with rejection-associated inflammation versus non-rejection-associated inflammation in full thickness skin (FTS), vascularized heterotopic skin-muscle-bone (SMB) composite allografts, and hind limb composite allografts are consistently and significantly different. In this model SMB can be engrafted under routine continuous immunosuppression; however, FTS will still be acutely rejected. Through multivariate analysis it was clearly observed that distinct immune signaling patterns mediate rejection in SMB versus FTS. Specific cytokines were observed as the primary drivers of these distinct patterns, and the biological functions of those cytokine ensembles were then elucidated and correlated with the numeric analysis to reveal that rejection-associated inflammation followed clearly different patterns in SMB and FTS [95] (Figure 2, paper in preparation).

## 5. Alternative and Experimental Methods for Detecting Rejection

Interest in finding a better means of detecting or predicting rejection has spawned a range of research approaches. Although these methods have not yet found widespread clinical adoption, the approaches and technical innovations are informative with regards to how challenges faced by the field are being overcome.

Utilizing little or no tissue data, the psychiatric analysis described by [96] concluded that although the features measured could be used to identify certain risk factors for rehospitalization, they were not predictive of rejection specifically. Rehospitalizations were due to a variety of causes, including immunosuppression-associated infection. One of the most predictive factors for rehospitalization included patient noncompliance with medication instructions.

A significant amount of ongoing research is being invested in finding genetic markers for rejection. The most promising results to date have come from [97] showing correlation between miRNA coding for cytotoxic proteins and rejection as well as [98] showing strong correlation between donor gene fragments in circulating blood and the progression of rejection. However, in the presented results there is a high degree of variance in key metrics measured, and the detection of rejection is thought to occur at the onset of graft damage. This may eventually provide an improvement over current clinical standards by reducing unnecessary biopsies and may eventually become a platform for more advanced miRNA-based analytical methods. Additional work in the area of genetic rejection detection has been done by [99, 100].

Cellular analysis is perhaps the most popular alternative approach to assessing rejection. A large number of biomarkers have been identified and catalogued [101] however, *in-vivo* most biomarkers suffer from high false positive rates or are not cost-effective to assess. For kidney transplant cases, [102] describes a method that is a reliable indicator in about 62% of studied cases. [103] identifies cells associated with rejection in circulating blood, but like [98], these cells provide limited predictive value beyond what may be achieved by pathologist examination of a biopsy.

Doppler tissue imaging as described by [104] may eventually provide a noninvasive alternative to heart biopsy. As described, the system is capable of providing 82% sensitivity and 53% specificity, although it does not confer predictive power.

Significant recent advances in proteomic analysis have been made by [105] who proposed a breath-test for heart transplant rejection that is capable of providing 71.4% sensitivity and 62.4% specificity. Excellent performance in predicting corneal transplant rejection was shown in [106] with the application of linear discriminant analysis to selected cytokines, reinforcing the potential clinical or diagnostic utility of computational and statistical inference methods.

## 6. Computational and Statistical Inference Literature

The analysis of systems that contain multiple dependent variables, unknown influencing factors, and context dependence presents an especially challenging problem to traditional methods of analysis such as ANOVA or other univariate methods of analysis. To elucidate the actual behavior of complex systems, and to build models with predictive power, more advanced methods of computational and statistical analysis are required. Concise and thorough coverage of the statistical inference and modeling methods that are extensively used in medicine and computational methods of biological analysis is given in [107–110]. Both discriminative and generative methods are important analytical tools in analyzing biological data. Discriminative methods are often able to produce classifiers that have superior performance in predicting class membership than their generative counterparts; however, generative methods allow data to be generated from the model, effectively allowing *in silico* simulation of system behavior through changes in model parameters. Agent-based models provide a means of understanding complex phenomenon by simulating the behavior of actors within the system, a technique that holds promise for demystifying many biological processes where simulation results can be appropriately constructed, evidentially linked to the biological reality, and experimentally verified. The construction and analysis of this class of computational models are discussed in [111, 112].

Many of the most promising methods and approaches that have the potential to improve the widespread adoption of VCA are at the intersection of medicine, immunology, mathematics, and computer science. By leveraging the strengths and capabilities of each field to solve problems that have been resistant to analysis in another, more rapid progress can be made in delivering novel and clinically relevant findings, diagnostics, or therapeutic compounds.

Approaches that take a cross-disciplinary approach and seek to synthesize the strengths of diverse fields, such as mathematics, computer science, and immunology, are providing new methods and insights that may help to advance the state of the art as well as the development of novel and clinically relevant technologies or therapies for VCA.
